# Effect of Contact Point of Wire Ring on Cooling Behavior during Stelmor Cooling

**DOI:** 10.3390/ma15228262

**Published:** 2022-11-21

**Authors:** Joong-Ki Hwang

**Affiliations:** School of Mechatronics Engineering, Korea University of Technology & Education, Cheonan 31253, Republic of Korea; jkhwang@koreatech.ac.kr; Tel.: +82-041-560-1642

**Keywords:** Stelmor, steel wire rod, contact point, hotspot, uniform cooling

## Abstract

The influence of the contact point of wire rod on the inhomogeneity of cooling behavior within wire ring was investigated to reveal the fundamental cooling mechanism of wire rod during the Stelmor cooling process. A hotspot, a relatively high-temperature region within wire ring compared with other regions, was generated in both the central (WR_c_) and edge (WR_e_) regions of the wire ring. The WR_e_ exhibited hotspots regardless of ring configuration. Meanwhile, the WR_c_ exhibited hotspots with an inline arrangement; otherwise, no hotspot occurred in the WR_c_ with a staggered arrangement. Compared with the middle regions of the wire ring, hotspots easily occurred at both the WR_c_ and WR_e_ due to the low-contact angle of the two wire rings. Moreover, the possibility of hotspot formation increased with increasing wire diameter due to the high-contact area and load caused by the weight of the wire rod. This is the primary reason why the WR_c_ with a large diameter had hotspots despite the large ring pitch. Three solutions were suggested to improve the homogeneity in the mechanical properties within wire ring.

## 1. Introduction

Most hot-rolling steel wire rod mills employ a Stelmor-type cooling system [1,2] because it can produce various kinds of wire rod products with a cost-effective, simple cooling line due to its wide spectrum of cooling rates (CR) [3,4]. This cooling system increases the CR of the workpiece using forced air driven by several fans and decreases the CR through compartmentalized insulated covers.

**Figure 1:**
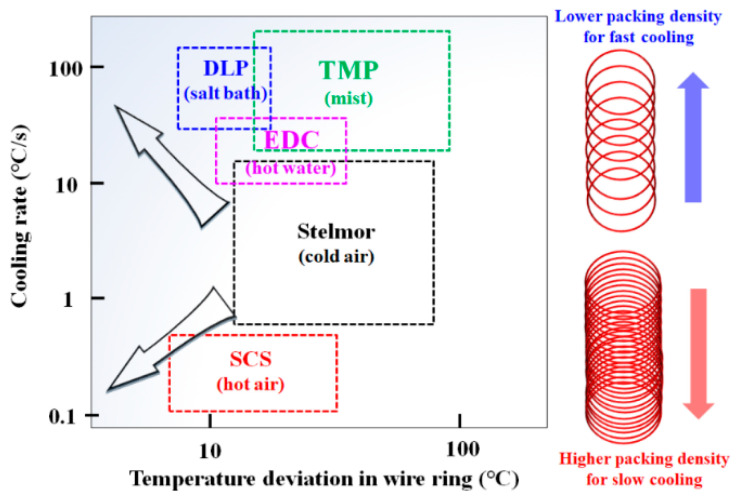
Schematic description of development trend in steel wire rod cooling system [6,7,8,9,10,11,12].

However, wire rod mills that adopt a Stelmor-type cooling system have three major concerns to be resolved: lack of accelerated cooling capacity to manufacture high-carbon steels with a large diameter [5], lack of retarded cooling capacity to manufacture high-alloyed steels without causing undesired martensite and bainite microstructures [6], and insufficient uniform cooling capacity to manufacture products while having homogeneous material properties in the wire ring [7]. To overcome these drawbacks of the Stelmor cooling system, there are two typical approaches adopted in the industry. First, several advanced wire rod mills have attempted to change the coolants from cold air to salt bath (DLP, Direct in-Line Patenting) [8,9], hot water (EDC, Easy Drawing Conveyor) [6,10], mist (TMP, Toa Mist Patenting) [11], and hot air (SCS, Slow Cooling System) [12], as shown in Figure 1. The DLP, EDC, and SCS processes are used in the industry due to the improved homogeneity of material properties within wire ring as well as control of the CR. Secondly, many wire rod mills adopted an airflow control system along the transverse direction of wire ring (TD_wr_) for uniform cooling as well as for increasing the cooling ability [13,14,15,16,17].

Between the two approaches mentioned above, the second approach is a cost-effective way to achieve uniform cooling within wire ring, although it is not as effective as the coolant change approaches. Therefore, during the past few decades, numerous studies have been conducted regarding the uniform cooling of wire ring during the Stelmor-type cooling process without changing the coolant. In the 1980s, Hanada et al. [18] set up an offline Stelmor simulator to determine the optimum working conditions for uniform cooling of a workpiece before installing the wire rod cooling system in a real plant. They increased the uniform cooling capacity along the TD_wr_ using an air velocity control damper system. Lindemann and Schmidt [19] evaluated the heat transfer phenomenon within wire ring based on a mathematical model that considered the geometric complexity of wire ring. Nobari and Serajzadeh [20] reported a mathematical model using the finite element method to analyze temperatures in wire ring, particularly at the core and outer surface regions of wire rod. Fang and Lin [13] reported an online diagnostic system of the temperature for wire rod cooling. They decreased the deviation in the tensile strength (TS) of wire ring by optimizing the distribution of the airflow rate along the TD_wr_ based on the temperature measurement system. Hong et al. [21] used three-dimensional commercial software for predicting the temperatures of wire ring during cooling. They showed temperature variations between the lower-layer and upper-layer of wire rings as well as between the central region (WR_c_) and edge region (WR_e_) of wire ring.

**Figure 2:**
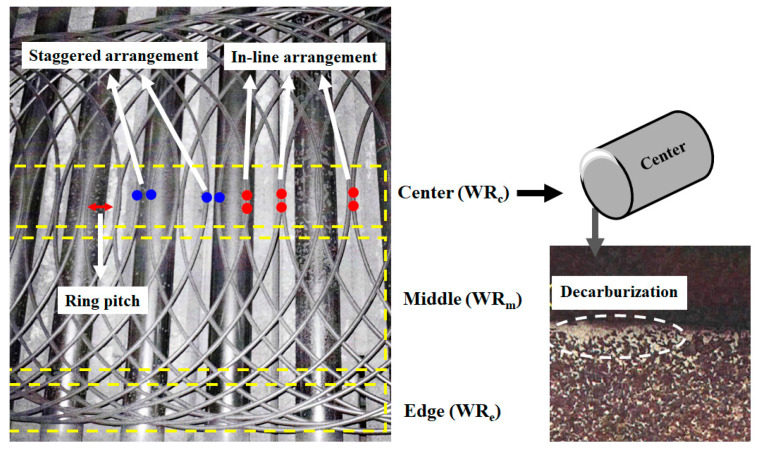
Configuration of wire ring on a Stelmor cooling system and microstructure of wire rod with local decarburization at central region of wire ring.

Recently, Hwang [22] revealed the cooling phenomena of wire ring during Stelmor cooling based on its complex geometrical structure. He provided that the variations in the material properties within wire ring were dependent on wire ring pitch (*P_wr_*) or wire packing density (WPD), as shown in Figure 2. However, nowadays, deviations in the mechanical properties and microstructure of wire rods with large diameters have become an issue in wire rod mills, although the *P_wr_* of wire rods with large diameters is larger than that of wire rods with small diameters. For example, a low TS or decarburization appeared in a WR_c_ with a large diameter, as shown in Figure 2. It is known that low TS and/or decarburization in plain carbon steels are indicators of slow cooling [7,23]. This cooling phenomenon of wire ring during Stelmor cooling cannot be explained using the *P_wr_* and WPD approaches. Typically, a low TS and local decarburization within wire ring should be generated in a WR_e_ with a small diameter according to the *P_wr_* and WPD approaches. From the author’s experience, this unexpected cooling behavior and microstructure in a WR_c_ with a large diameter were related to the contact point of the wire ring during cooling. However, no study has been conducted on the influence of the contact point of the wire ring on its cooling behavior during the Stelmor-type wire rod cooling process.

Therefore, to reveal the more fundamental cooling mechanism of wire ring and to reduce the variation of the material properties of wire ring in the Stelmor cooling system, the author primarily focused on the effect of the contact point of the wire ring on the inhomogeneity of the CR in the wire ring. In addition, the concept solutions for increasing the homogeneity of material properties in wire ring are suggested in an applicable way. An offline cooling simulator and geometric mathematical analysis of wire ring were used to reveal the influence of the contact point within the wire ring on the cooling behavior.

## 2. Theoretical Background

During the Stelmor wire rod cooling process, the nonuniform cooling behavior of the wire ring within the region is attributed to the difference in WPD along the TD_wr_ [22,24,25,26,27]. In other words, WPD increased from the WR_c_ to WR_e_, as shown in Figure 2. In particular, the author [22] calculated the WPD of a wire ring on a conveyor roller during Stelmor cooling with the following assumptions: (i) the overall ring shape is a perfect circle, (ii) *P_wr_* is constant in the entire process, indicating that the final rolling velocity (*V_roll_*), conveyor roller velocity (*V_conv_*), and diameter of wire ring (*D*) are unchanged during the process, and (iii) the relative volume of the wire rod is equivalent to the two-dimensional projection area of the three-dimensional wire rod.

**Figure 3:**
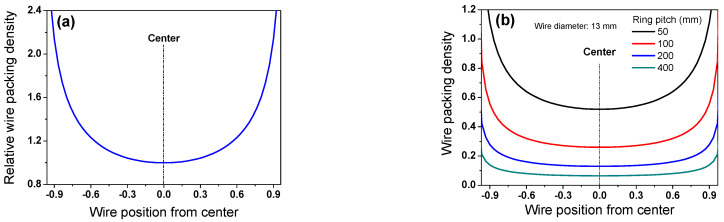
(**a**) Relative wire packing density along TD_wr_ and (**b**) comparison of wire packing density as a function of ring pitch during Stelmor cooling.

Figure 3a depicts the calculated WPD based on these assumptions. The WPD in the middle region (WR_m_) and WR_e_ are higher than that in the WR_c_. As expected, the WPD increased with a decrease in *P_wr_*, as shown in Figure 3b. *P_wr_* can be mathematically determined using the following equation:

(1) $$\;P_{wr}=\frac{\pi DV_{conv}}{V_{roll}}$$

For a more in-depth understanding of the Stelmor cooling process in real plants, *V_roll_* was derived as a function of wire diameter (*d*) with the assumption of constant mill productivity ($\dot{Q}$) regardless of *d* as follows:

(2) $$\dot{Q}=AV_{roll}=\frac{\pi d^{2}}{4}V_{roll}=C$$

(3) $$V_{roll}=\frac{4C}{\pi d^{2}}$$

where *C* means the constant value that varies with wire rod plants. *V_roll_* exhibited an inverse relationship with *d*^2^. Combining Equations (1) and (3), *P_wr_* can be expressed as follows:

(4) $$P_{wr}=\frac{\pi^{2}d^{2}DV_{conv}}{4C}$$

**Figure 4:**
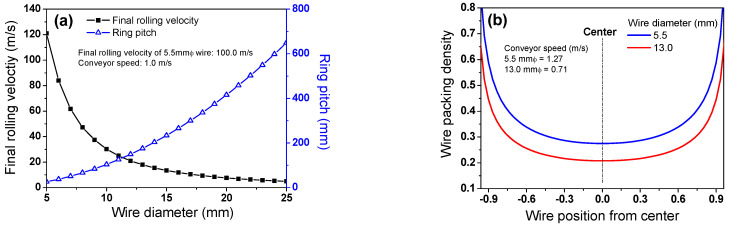

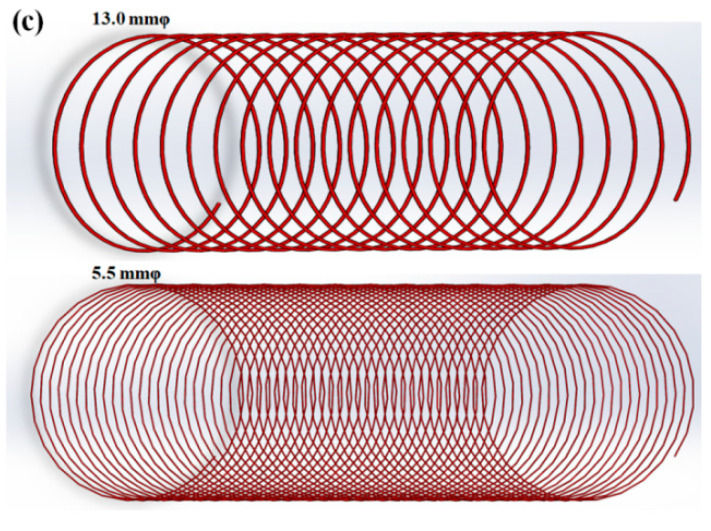
(**a**) Variations in final rolling speed and ring pitch with wire diameter and (**b**,**c**) comparison of wire packing density with wire diameter in a typical wire rod mill.

*P_wr_* increased in proportion to the square of *d*. To determine the *C* in a wire rod mill, the *V_roll_* of a wire rod with a *d* of 5.5 mm was set at 100 m/s, which is the general case in industries. In this case, *V_roll_* and *P_wr_* have a relationship with *d* under the assumption of constant *D* and *V_conv_*, as shown in Figure 4a. This implies that *P_wr_* is highly related to *d*. In real plants, *V_conv_* tended to decrease with increasing *d* because of the limited length of a Stelmor conveyor. In other words, the *V_conv_* of a wire rod with a large diameter should be decreased to finish the cooling within the Stelmor cooling conveyor due to the slow CR of a large wire rod. Although *V_conv_* was reduced with increasing *d*, the WPD of a small wire rod was higher than that of a large wire rod, as shown in Figure 4b,c. This is because *P_wr_* is proportional to *d*^2^ and *V_conv_*, as shown in Equation (4).

Meanwhile, based on the results of the WPD along the TD_wr_ (Figure 3a), a higher airflow rate is forced on the WR_e_ rather than the WR_c_ and WR_m_ using the airflow rate control damper system in real plants [13,14]. Moreover, the difference in the airflow rate between the WR_c_ and WR_e_ increased with decreasing *d* [25] because the WPD increased with decreasing *d* (Figure 4b,c).

## 3. Experimental Procedures

**Figure 5:**
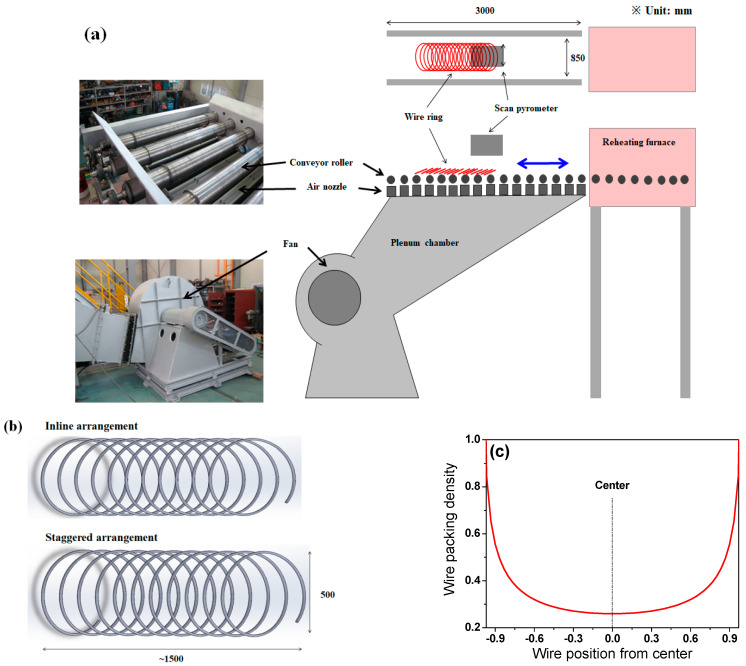
(**a**) Photograph and schematic of the cooling simulator, (**b**) two types of wire ring configurations, and (**c**) calculated wire packing density of the specimen used in this test.

A Stelmor simulator was used to reveal the cooling behavior of a wire ring with the ring configuration. The simulator consisted of an electrical reheating furnace with atmospheric gas control, a roller table with speed control, an air fan with airflow rate control, and two types of wire ring specimens, as shown in Figure 5.

A steel wire rod with a 13 mm diameter was chosen for the experiment because small workpieces are more sensitive to other experimental conditions during temperature measurements. Due to the limited scale of the simulator (Figure 5a), *D* was chosen as 500 mm, as shown in Figure 5b. To understand the influence of the contact point or ring configuration of the wire ring on the CR, two types of ring specimens with inline and staggered ring configurations were prepared (Figure 5b). Plain high-carbon steel, SWRS82B, was selected as a test metal. Its chemical composition and physical and mechanical properties of the hot-rolled wire are listed in Table 1 and Table 2, respectively. Figure 5c shows the calculated WPD along TD_wr_ of the specimen used in this test. The specimen was heated to 1050 °C in the reheating furnace to austenitize the specimen under N_2_ gas and then held at 930 °C for 5 min to simulate the laying head temperature in real plants. Subsequently, it was cooled by forced air with a velocity of 37 m/s driven from the blower under the conveyor roller, as shown in Figure 5a.

Considering the round-shaped, small wire, a scan pyrometer that had a wavelength of 0.9 μm was used for temperature measurement. It scanned the wire ring at 2048 points using a CCD linear array along TD_wr_. The measuring distance from the pyrometer to wire ring was approximately 1.0 m. The accuracy of temperature measurement in this pyrometer was ±1.0% of the measured value. Under similar working conditions as the offline cooling simulation, the temperatures of the SWRS82B wire rod were obtained in a wire rod mill using a FLIR thermocamera that had a wavelength of 7.5–14 μm (Table 3). The resolution of the thermocamera was 640 × 480 pixels; the measuring distance of the thermometers and wire ring was approximately 1.5 m. The ambient temperature was approximately 24 °C in the laboratory where the simulator was located and approximately 27 °C in a wire rod mill.

A portable velocity meter with pitot static tube was used to measure the air velocity at nozzle. The velocity accuracy of the equipment was ±0.25% of measured value. The microstructures were compared using scanning electron microscopy (SEM) that was operated at 20 kV. SEM observations were performed on the cross-section of a specimen perpendicular to the wire rod axis.

## 4. Results and Discussion

### 4.1. Experimental Results

**Figure 6:**
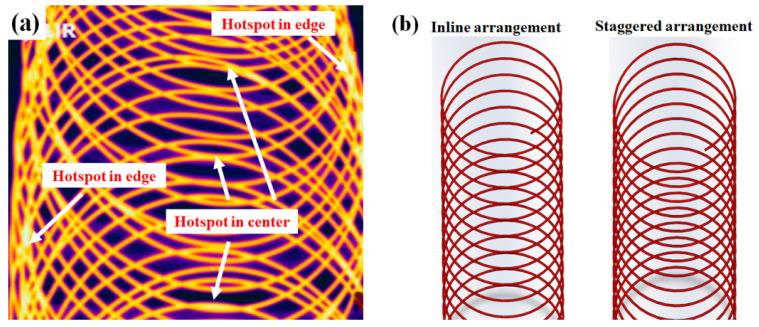
(**a**) Thermal image using the thermocamera in a wire rod mill and (**b**) schematic of two types of ring configuration on the conveyor roller during Stelmor cooling.

Figure 6a shows a representative thermal image measured with the thermocamera in the wire rod mill. The high-temperature region, hereafter called the hotspot, occurred in both the WR_c_ and WR_e_. A hotspot is defined as a high-temperature region in the wire ring compared with other regions of the wire ring. From the author’s experience, a thermocamera tends to overestimate the temperature in high-packing regions, such as the WR_e_, and underestimate the temperature in low-packing regions, such as the WR_c_. Based on this, we could predict that the temperature in the WR_c_ was relatively high. In particular, the temperature in the WR_c_ with the inline arrangement was higher than that of the wire ring with the staggered arrangement.

**Figure 7:**
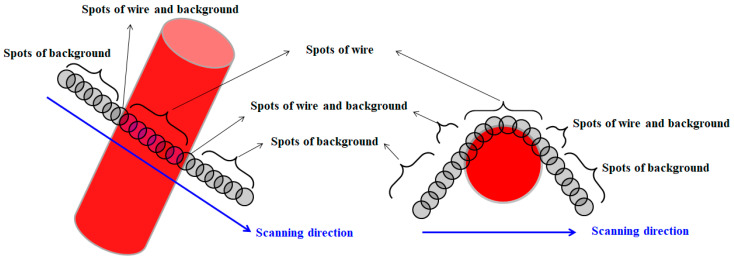
Schematic of temperature measuring method of wire rod using a scan-type pyrometer during Stelmor cooling.

**Figure 8:**
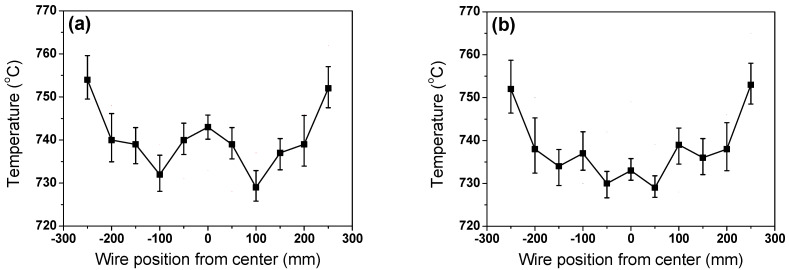
Comparison of measured temperatures of wire ring along TD_wr_ with (**a**) inline and (**b**) staggered arrangements in the Stelmor simulator.

Figure 7 schematically explains the temperature measurement method using a scan-type pyrometer. It is not easy to determine wire rod temperature due to round shape of the wire and small diameter [28]. To detect the real wire rod temperature from the mechanical structures of the simulator, five consecutive high temperatures were measured and recognized as the temperature of the specimen. In addition, the average temperature was determined as the wire rod temperature in this region. Figure 8 shows a comparison of the measured temperature profiles of the specimen between the inline and staggered configurations. The temperatures in the WR_e_ and WR_m_ were similar between the two ring configurations; however, the temperature in the WR_c_ was significantly different between the two ring configurations. The WR_c_ with the inline arrangement was much higher than that with the staggered arrangement, which was consistent with the thermal image obtained by thermocamera.

**Figure 9:**
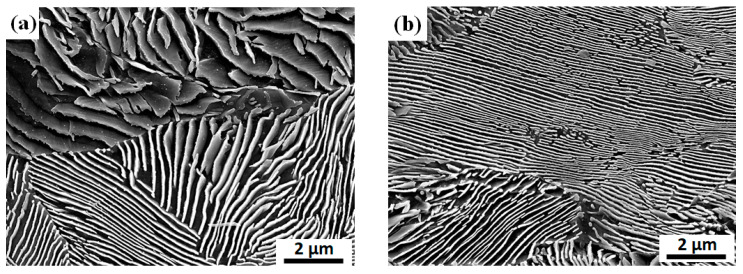
Comparison of representative microstructure at the central region of wire ring with (**a**) inline and (**b**) staggered arrangements.

To analyze the impact of wire ring configuration on cooling behavior in more detail, the microstructures in the WR_c_ between the inline and staggered arrangements were compared. Figure 9 compares the representative microstructure at the WR_c_ of the wire ring with inline and staggered arrangements. The specific experimental conditions are described in Table 3 and Figure 5 in the experimental section. Lamellar structures of ferrite and cementite appeared in both wires. However, the interlamellar spacing of the wire with the staggered arrangement was slightly smaller than that of the inline arrangement because the CR of the WR_c_ with the staggered arrangement was higher compared with that of the inline arrangement (Figure 8). The refinement of the pearlitic microstructure increases the resistance to dislocation glide because of the interfaces between ferrite and cementite act as barriers to dislocation movement. That is, the decrease of pearlite interlamellar spacing leads to an increase in the strength of pearlitic steels, indicating that the Hall–Petch type strengthening effect can be applied in pearlitic steels. Meanwhile, it is generally accepted that the interlamellar spacing of pearlitic steels decreases as the CR increases [29,30]. For example, the wire rod industry for pearlitic steel products conducts a patenting heat treatment to obtain the fine pearlitic structure of wires by performing the phase transformation from austenite to pearlite at the nose temperature in a continuous cooling transformation diagram of steel [8]. The patenting heat treatment is one of the step cooling processes: strong cooling in the early stage and isothermal cooling at nose temperature in a continuous cooling transformation diagram of a metal using a molten salt or lead. Overall, the WR_c_ of the wire ring with inline arrangement exhibited coarse lamellar structures compared with the wire ring with staggered arrangement due to the low CR of the wire, as shown in Figure 9.

Although *P_wr_* and WPD were similar (Figure 5b,c), the CRs of the wire rings were different depending on the ring configurations. This result provided important information on Stelmor cooling. Selecting the right ring configuration is essential to increase the uniform cooling ability within the wire ring. The ring configuration with the staggered arrangement is recommended for uniform cooling of the wire rod because more intensive air is imposed on the WR_e_ compared with the WR_c_ in real plants using a flow rate control damper system to uniformly tailor the material properties of the wire rod [15,16]. In such a case, the WR_c_ with an inline arrangement cooled quite slowly compared with the other regions, leading to decarburization and low TS in this region, indicating that the ring configuration, and *P_wr_* is an important parameter for controlling the cooling behaviors of the wire ring in the Stelmor-type cooling system. In other words, the CR of the wire ring was strongly dependent on the ring configuration (Figure 8); the CR determined the distribution in microstructure (Figure 9) and mechanical properties in the wire ring. Based on this result, the makers in wire rod mills should focus on the configuration of wire ring to reduce variation in product quality.

### 4.2. Geometric Analysis of Contact Points in Wire Ring

The unexpected characteristic of the present study can be summarized as follows: (i) a hotspot was generated in the WR_c_ (Figure 6 and Figure 8) although the WPD at the WR_c_ was lower than that of the other regions (Figure 3a), (ii) hotspots rarely occurred in the WR_m_, otherwise, the WR_c_ and WR_e_ exhibited hotspots, and (iii) decarburization appeared in the WR_c_ with a large diameter (Figure 2) despite the low WPD compared with the wire rod with a small diameter (Figure 4b).

**Figure 10:**
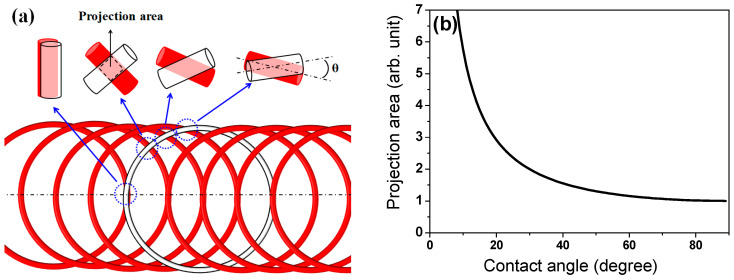

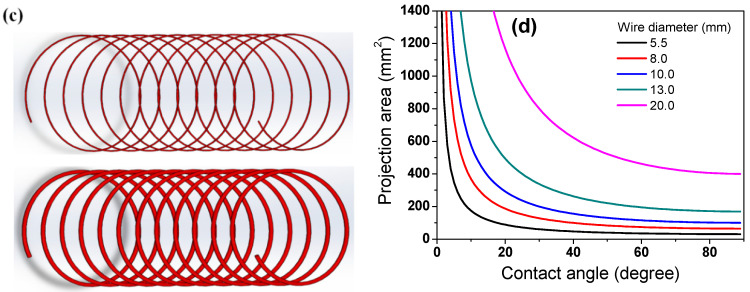
(**a**,**c**) Schematic and (**b**,**d**) calculated projection area of wire rod with contact angle and wire diameter.

Based on both the WPD approach and general knowledge of the heat transfer mechanism, it is difficult to understand the cooling phenomena observed above. Therefore, to comprehend the cooling behavior of the wire rod, the contact point of the wire ring was analyzed. The projection area (*A_p_*) at the contact point was calculated using simple mathematics as illustrated in Figure 10. *A_p_* decreased with an increasing contact angle (*θ*) of the two wire rods (Figure 10a,b). In addition, *A_p_* increased with *d* (Figure 10c), which can be mathematically represented as follows:

(5) $$A_{p}=\frac{d^{2}}{\sin\theta }$$

Figure 10d compares the *A_p_* as a function of *d* and *θ*. The *A_p_* increases as *d* increases and *θ* decreases.

**Figure 11:**
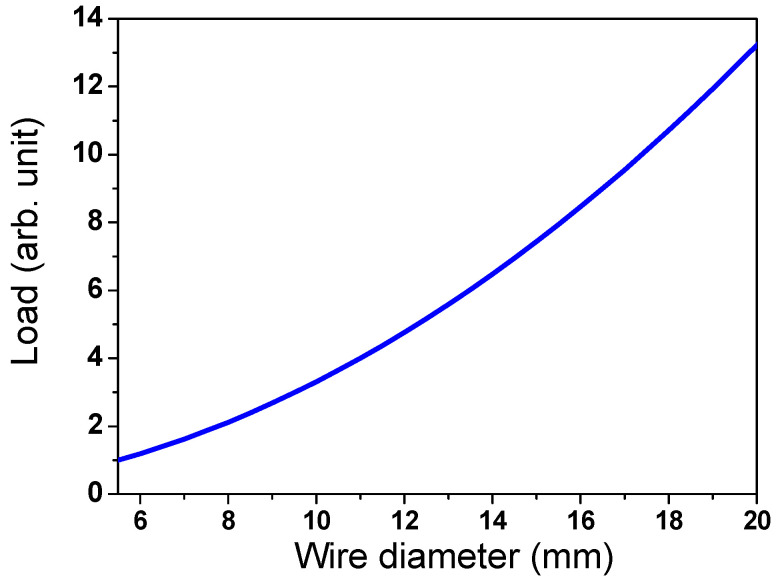
Variation of load by weight of upper wire ring as a function of wire diameter.

The load (*L_wr_*) at the contact point of the wire ring by the weight of the upper wire rod was calculated using the following equations:

(6) $$L_{wr}=\rho V_{wr}g$$

(7) $$V_{wr}=\frac{\pi d^{2}}{4}\pi D$$

where *ρ* and *V_wr_* are the density and volume of the wire rod, respectively. *L_wr_* increased in proportion to the square of *d*, as shown in Figure 11. It should be noted that the number of contact points per wire ring was not considered to calculate the *L_wr_* in this study. Based on *A_p_* and *L_wr_*, the possibility of hotspot formation within the wire ring, which is called a hotspot indicator (*I_hs_*) hereafter, was defined as follows:

(8) $$I_{hs}=L_{wr}A_{p}=\rho g\pi^{2}D\frac{d^{4}}{4\sin\theta }$$

**Figure 12:**
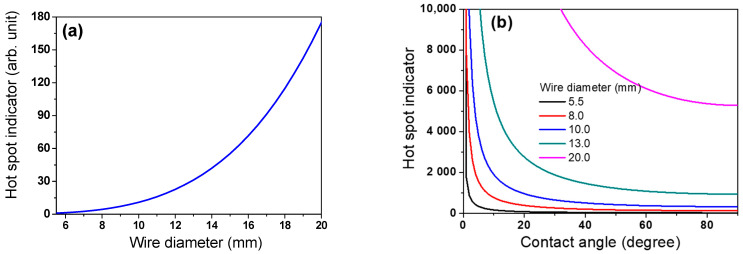
Calculated hotspot indicator as a function of (**a**) wire diameter and (**b**) both wire diameter and contact angle.

**Figure 13:**
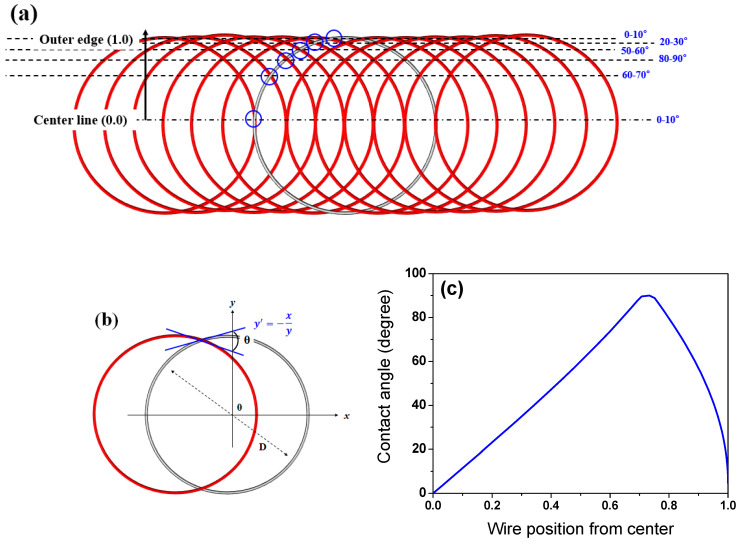
(**a**,**b**) Schematic of contact angle of wire ring and (**c**) calculated contact angle of wire ring along TD_wr_ during Stelmor cooling.

**Figure 14:**
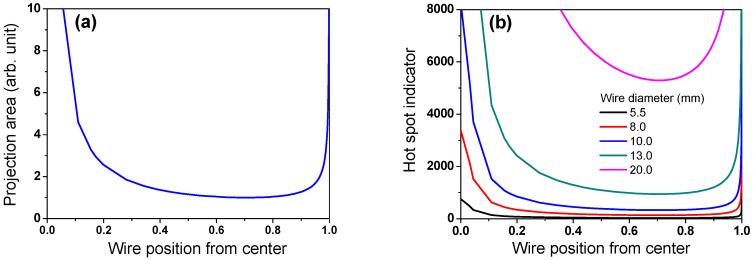
Calculated (**a**) projection area of wire ring with wire position and (**b**) hotspot indicator as a function of wire diameter and wire position during Stelmor cooling.

Hotspots were generated at the contact point of the wire ring (Figure 6). According to Equation (8), the hotspot was stronger with increasing *A_p_* and *L_wr_* because the heat dissipation of the wire ring by convection and radiation was retarded with increasing *A_p_*, and the contact point hardly shifted during the entire cooling process with increasing *L_wr_*. That is, the *I_hs_* increases as *d* increases and *θ* decreases, as shown in Figure 12 and Equation (8). Figure 13 calculated the *θ* along the TD_wr_. When the wire ring was assumed to be a perfect circle (Figure 13b), *θ* increased from the center to a certain distance and then decreased from the WR_m_ to WR_e_, as shown in Figure 13c. In other words, *θ* increased up to 0.707 of the ring radius from the center and decreased thereafter. For example, *θ* is 0° at the WR_c_ and outer WR_e_, and *θ* is 90° at 0.707 of the ring radius. Combining the above calculation and Equation (8), the *I_hs_* was induced along the TD_wr_ (Figure 14). When the hotspot was generated in the WR_c_ and WR_e_, the strength of the hotspot was high, and, in particular, the wire diameter was high. This is the primary reason for the formation of the hotspot in the WR_c_ with a large diameter despite the low WPD in this region. In addition, the result explained the high temperature at the WR_c_ with the inline arrangement and the low temperature at the WR_c_ with the staggered arrangement, as shown in Figure 8. In the industry, hotspots are easily observed in wire rod with a large *d* rather than wire rod with a small *d*, although the WPD was lower as the *d* increased (Figure 4). This phenomenon can be explained using this *I_hs_* approach (Figure 14b). As *d* increased, the CR was retarded as the *A_p_* increased. Furthermore, with increasing *d*, the contact points hardly shifted in conveyor rollers during the entire cooling process due to the high *L_wr_*. Overall, the cooling behaviors within the wire ring in Stelmor cooling can be evaluated by analyzing the contact point of the wire ring.

### 4.3. Effect of Ring Pitch and Solutions for Improving Uniformity in Wire Ring

**Figure 15:**
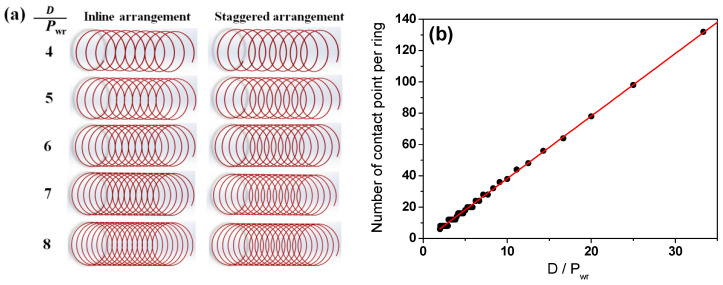
(**a**) Schematic and (**b**) calculated number of contact points in wire ring as a function of ring pitch in Stelmor cooling.

*P_wr_* affects both the WPD and contact point of the wire ring as depicted in Figure 15. The number of contact points per wire ring (*N_cp_*) was calculated as follows:

(9) $$N_{cp}=3.9\left(\frac{D}{P_{wr}}\right)$$

**Figure 16:**
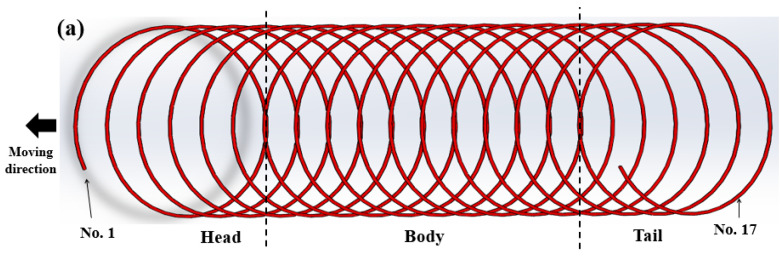

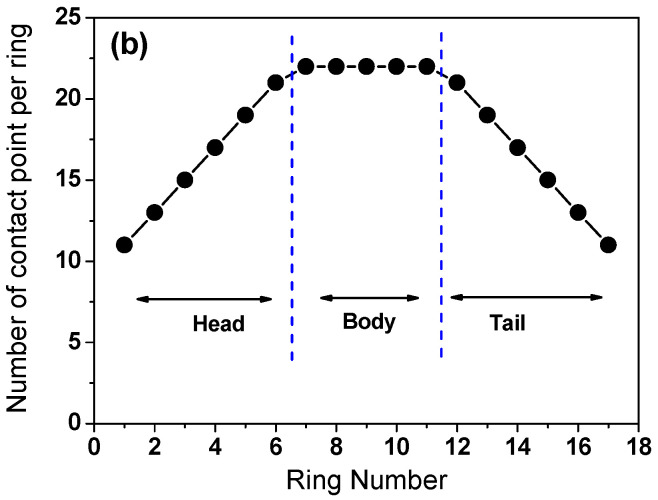
(**a**) Schematic and (**b**) measured number of contact point in wire ring along the moving direction of wire ring on a Stelmor conveyor roller.

As expected, *N_cp_* has an inverse relationship with *P_wr_*, indicating that a large *P_wr_* induced a more uniform cooling within the wire ring due to both the low WPD and small *N_cp_*. Similarly, both the head region and tail region were cooled more uniformly compared with the body region, as shown in Figure 16, due to the low *N_cp_*.

Three concept solutions were induced to reduce the variation in the CR, microstructure, and mechanical properties of the wire ring product during Stelmor cooling from the present results with the aid of experiments using the Stelmor simulator and contact point analysis of the wire ring.

(i) It is necessary to avoid the contact points in the WR_c_ and WR_e_ during cooling. In particular, the wire configuration with an inline arrangement should be avoided by controlling *P_wr_* using *V_conv_* because the CR in contact points of the wire ring in the WR_e_ was tailored by the airflow control damper system in the industry [21,31]. However, no equipment or process has been reported for cooling rate control at the WR_c_ in general wire rod plants. In addition, the maximization of *P_wr_* within the mill capacity can reduce *N_cp_*.
(ii) Shifting techniques of contact points of the wire ring on a conveyor roller are strongly necessary. For example, the steps between the conveyor roller and differentiated conveyor roller speed can shift the hotspot in each conveyor roller zone.
(iii) Changing the direction of the air nozzle can reduce the hotspot effect of the wire ring. For example, oblique air blowing increases the CR of the contact regions in the wire ring. In the same vein, immersing the wire ring in hot water (EDC) and salt bath (DLP) can increase the CR of the contact point of the wire ring. Meanwhile, mist cooling (TMP) could increase the inhomogeneity of the material properties of the wire ring due to the difficulty in tailoring the cooling behavior at the contact point of the wire ring.

From the perspective of operation in real plants, these three suggestions are helpful in designing working conditions in wire rod mills to manufacture high-quality wire rod products.

## 5. Conclusions

Based on a comparative study of the influence of ring configuration on the thermal behavior of wire rod and geometric analysis of wire ring in Stelmor-type cooling, the following conclusions were derived:

1. A hotspot was generated in both the WR_c_ and WR_e_. The WR_e_ exhibited hotspots regardless of ring configuration. Meanwhile, a hotspot appeared in the WR_c_ with an inline arrangement; otherwise, no hotspot occurred in the WR_c_ with a staggered arrangement.
2. Compared with the WR_m_, hotspots easily occurred at both the WR_c_ and WR_e_ due to the low contact angle of the wire ring. Moreover, the possibility of hotspot formation increased with increasing wire diameter due to the large contact area and load caused by the weight of the wire rod, although *P_wr_* increased with increasing wire diameter. This is the primary reason why the WR_c_ with a large diameter showed hotspots despite the large *P_wr_*.
3. The number of contact points per wire ring increased as *P_wr_* decreased, indicating that the retarded cooling at the contact point of the wire ring depended on *P_wr_*. The ring configuration as well as *P_wr_* needs to be controlled for uniform cooling of the wire rod because the inline-arranged wire ring generated hotspots at the WR_c_ despite the large *P_wr_*.
4. Three basic solutions were suggested to reduce the inhomogeneity in the mechanical properties of the wire ring during Stelmor cooling: (i) avoiding the inline arrangement of the wire ring, (ii) shifting the hotspot in the wire ring during the entire working process, and (iii) employing an oblique nozzle. For instance, the conveyor speed in each zone should be changed during the process to shift the point of the hotspot in the wire ring; at the same time, the shifted hotspot should be effectively cooled by controlling the air-blowing direction using the nozzle. In addition, the monitoring system of the wire ring is necessary to avoid and control the inline arranged wire ring. These suggestions are helpful for setting cooling conditions in wire rod mills to manufacture high-quality wire rod products.

## Figures and Tables

**Table 1:** Chemical compositions of SWRS82B steel (wt.%).

C	Mn	Si	P	S	Fe
0.82	0.75	0.20	<0.01	<0.01	Bal.

**Table 2:** Physical and mechanical properties of SWRS82B steel.

Parameter	Value
Density at 25 °C	7751 kg/m^3^
Heat capacity at 25 °C	0.46 kJ/kg/°C
Tensile strength	1165 MPa
Total elongation	12.1%
Reduction in area	41.2%

**Table 3:** Working conditions and thermometer specifications for temperature measurements.

Application	Temperature Measuring Systems			Experimental Conditions			
	Instrument	Wavelength (μm)	Emissivity	Diameter of Wire Rod (mm)	Austenizing Temperature (°C)	Discharging Temperature (°C)	Average Air Velocity (m/s)
Offline simulator	CHINO pyrometer	0.9	0.89	13	1050	930	37
Wire rod mill	FLIR thermocamera	7.7–14	0.88	13	1100	900	37

## Data Availability

Not applicable.

## Nomenclature

*A* *_p_*	projection area of wire rod in the contact point (m^2^)
*D*	diameter of wire ring (m)
*d*	diameter of wire rod (m)
*g*	acceleration due to gravity (m/s^2^)
*L* *_wr_*	load at the contact point of the wire ring by the weight of the upper wire rod (N)
*N* *_cp_*	number of contact points per wire ring
*P* *_wr_*	ring pitch of wire ring (m)
$\dot{Q}$	mill productivity (m^3^/s)
*V* *_conv_*	conveyor roller speed (m/s)
*V* *_roll_*	final rolling speed (m/s)
*V* *_wr_*	volume of the wire rod (m^3^)
*θ*	contact angle of the two wire rods (^o^)
*ρ*	density of the wire rod (kg/m^3^)
*Subscript*	
c	central region
e	edge region
m	middle region
hs	hotspot
wr	wire ring
